# Surgical Management of Lumbar Disc Herniation in Late Pregnancy Under General Anesthesia in the Prone Position: A Case Report

**DOI:** 10.7759/cureus.113834

**Published:** 2026-08-02

**Authors:** Kohei Okuyama, Masahiro Inoue, Mai Shimizu, Saki Taiji, Sumihisa Orita, Kazuhide Inage, Yasuhiro Shiga, Shoko Yamagishi, Kyongsuk Son, Natsuko Nozaki-Taguchi, Seiji Ohtori

**Affiliations:** 1 Orthopaedic Surgery, Chiba University, Chiba, JPN; 2 Orthopaedic Surgery, Funabashi Central Hospital, Funabashi, JPN; 3 Anaesthesiology, Chiba University, Chiba, JPN; 4 Anaesthesiology, SHOWA Medical University, Tokyo, JPN; 5 Anaesthesiology, Funabashi Central Hospital, Funabashi, JPN

**Keywords:** general anesthesia, lumbar discectomy, lumbar disc herniation, pregnant patient, prone position

## Abstract

Symptomatic lumbar disc herniation during pregnancy is rare, and surgery in the prone position during late pregnancy is challenging because of the risk of abdominal and aortocaval compression. A 42-year-old woman at 32 weeks of gestation presented with severe bilateral buttock pain, left radiculopathy, perineal sensory disturbance, and later bladder and bowel dysfunction due to L4-5 lumbar disc herniation. Emergency posterior lumbar discectomy was performed under general anesthesia in the prone position. A four-point support table combined with hammock-style abdominal suspension using elastic bandages and stockinettes allowed abdominal decompression and continuous cardiotocography monitoring. Her leg pain improved after emergence from anesthesia, and fetal status remained stable. She later delivered a healthy boy. Prone-position lumbar surgery may be considered in selected patients during late pregnancy when abdominal compression is avoided, fetal monitoring is maintained, and emergency obstetric intervention is available.

## Introduction

Low back pain is common during pregnancy, whereas symptomatic lumbar disc herniation (LDH) is rare, with an estimated incidence of approximately one in 10,000 pregnancies [1]. Most pregnant patients with LDH are treated conservatively with analgesics, rest, physical therapy, or epidural steroid injections. However, surgery is indicated when bladder and bowel dysfunction or progressive neurological deficits occur [2].

Surgical treatment during pregnancy requires consideration of gestational age, maternal hemodynamics, fetal status, anesthesia, and operative position. In late pregnancy, prone positioning is generally avoided because the enlarged uterus may cause abdominal and aortocaval compression [3,4]. Therefore, the lateral decubitus position is often selected in the third trimester [5,6]. However, posterior lumbar discectomy in the prone position is a standard and familiar procedure for spine surgeons and can provide rapid decompression when safely performed.

We report a case of emergency posterior lumbar discectomy performed in the prone position in a COVID-19-positive patient at 32 weeks of gestation. The main purpose of this report is to describe the positioning method, fetal monitoring, and perioperative management that enabled safe prone-position surgery during late pregnancy.

## Case presentation

A 42-year-old multiparous woman, gravida 2 para 1, at 32 weeks of gestation presented to our hospital with a two-day history of severe bilateral buttock pain and left lower-extremity pain. Because her symptoms worsened rapidly and she became unable to walk, she was admitted on the same day. Her medical history included gestational diabetes mellitus and a recent positive test for COVID-19.

**Figure 1:**
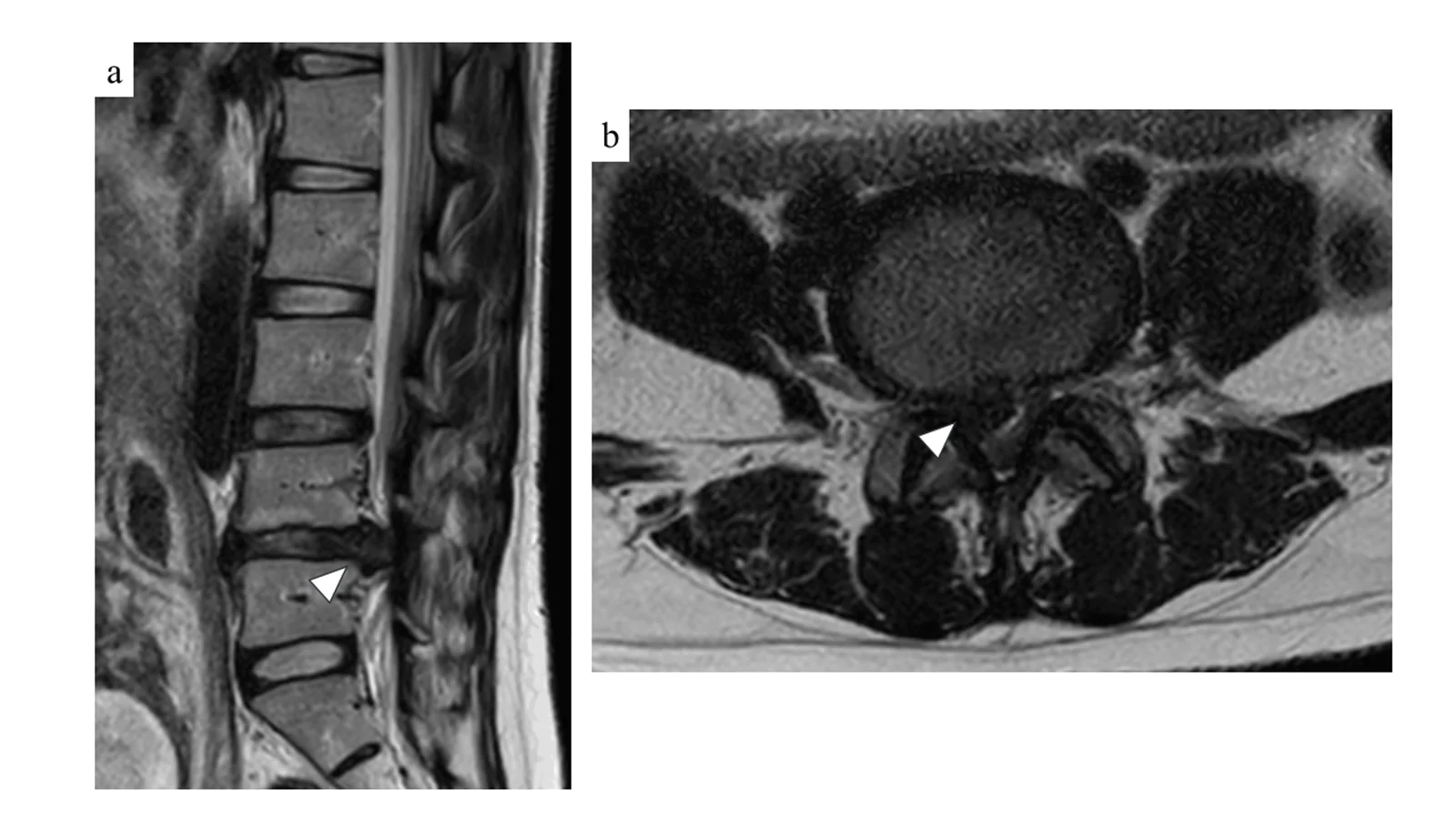
T2-weighted magnetic resonance images of the lumbar spine (a) Sagittal image showing a lumbar disc herniation at the L4–L5 level (arrowhead); (b) Axial image showing the herniated disc compressing the dural sac and adjacent nerve root at the same level (arrowhead).

Physical examination showed weakness of the left extensor hallucis longus, radicular pain along the left L5 dermatome, bilateral buttock pain, and sensory disturbance in the perineal region. Magnetic resonance imaging demonstrated L4-5 LDH occupying more than 50% of the spinal canal, with severe compression of the dural sac and the left L5 nerve root (Figure 1).

Conservative pharmacological treatment was initially attempted. However, bladder and bowel dysfunction developed on the third hospital day. After discussion among the orthopedic, anesthesiology, and obstetric teams, the treatment options and risks were explained in detail to the patient and her family. Cesarean delivery followed by spinal surgery was considered; however, the obstetric team did not recommend delivery at 32 weeks because of the risks associated with prematurity. Non-obstetric surgery during pregnancy was considered acceptable when required for maternal indications. Because urinary retention and bladder and bowel dysfunction suggested a risk of irreversible neurological deficit, emergency posterior lumbar discectomy under general anesthesia was planned with the fetus in utero. The prone position was selected because posterior lumbar discectomy in this position was the standard approach familiar to the operating spine surgeon and allowed direct exposure and rapid decompression. Decompression in the lateral decubitus position was not routinely performed in our practice and could have prolonged the procedure in this emergency setting. Therefore, the main issue was whether a stable prone position could be obtained without abdominal compression.

**Figure 2:**
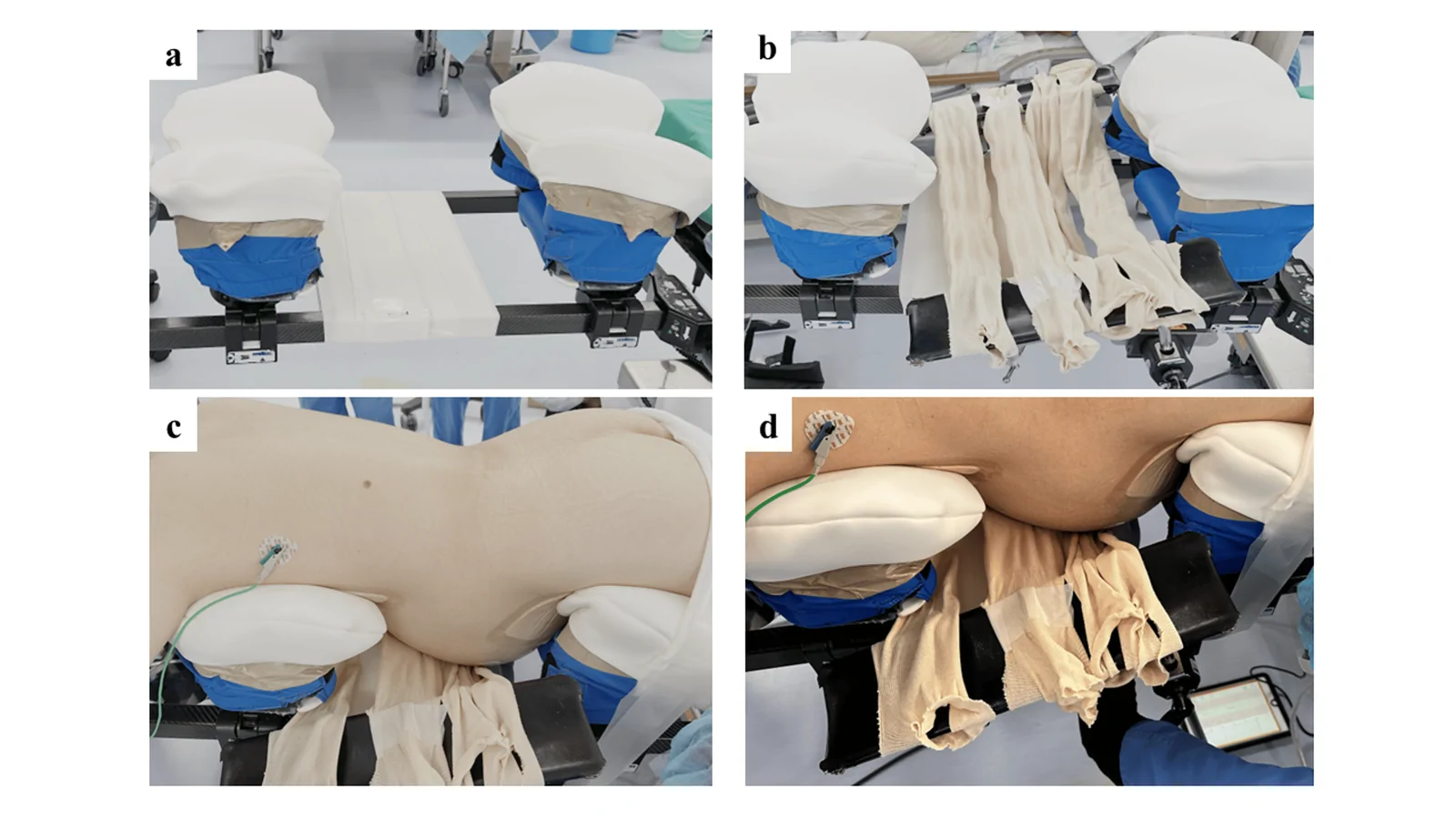
Techniques for ensuring safety in the prone position using a four-point support table a) Setup of the elastic bandage hammock for abdominal decompression; b) Reinforcement of the abdominal suspension with a stockinette layered over the initial elastic bandages; c) Final adjustment of the hammock tension after the induction of general anesthesia; d) Placement of a cardiotocography monitor by an obstetrician.

A four-point support operating table (Allen® Spine System, Allen Medical Systems, Acton, US) was used to support the chest and pelvis and to keep the abdomen free from direct pressure. However, this setting left the enlarged abdomen unsupported. Therefore, a hammock-like abdominal suspension was added using wide elastic bandages and stockinettes (Figure 2a).

The suspension was adjusted to avoid direct compression of the abdomen while preventing excessive downward displacement of the gravid uterus (Figures 2b, 2c). A small free space was maintained below the abdomen, and sufficient clearance was preserved to attach a cardiotocography (CTG) monitor (Figure 2d).　

During surgery, the fetal heart rate tracing was monitored by an obstetrician outside the operating room, and communication with the orthopedic and anesthesiology teams was maintained throughout the procedure.

A trial prone-position change under fetal heart rate monitoring was planned before induction to confirm positional tolerance. However, this was not feasible because the patient had severe pain despite intravenous fentanyl. General anesthesia was induced using a rapid-sequence technique in the supine position and was maintained with propofol-based total intravenous anesthesia.

Maternal blood pressure was actively managed to preserve uteroplacental perfusion. The target was a mean arterial pressure of at least 65 mmHg and approximately within 10-15% of the pre-induction baseline. The maternal blood pressure was 110/70 mmHg before induction and 95/55 mmHg after induction. Phenylephrine boluses and continuous norepinephrine infusion (0.05-0.1 μg/kg/min) were used. Fetal heart rate monitoring was continued from before induction until emergence.

Before surgery, a contingency plan was shared among all teams. If a non-reassuring fetal status occurred, the spinal procedure would be stopped immediately, the patient would be returned to the supine position, and emergency cesarean delivery would be performed. The necessary equipment was prepared, an obstetrician remained available in the hospital, and communication among the orthopedic, anesthesiology, and obstetric teams was maintained throughout the procedure.

Because the patient was positive for COVID-19, the operation was performed at our hospital under infection-control measures. After induction, the patient was carefully turned to the prone position. After prone positioning, maternal blood pressure decreased to 75/45 mmHg, and continuous norepinephrine infusion was started. The blood pressure subsequently stabilized at approximately 95/60 mmHg before incision. The CTG monitor remained in place, and a stable fetal heart rate pattern was confirmed before incision. No abnormal fetal heart rate pattern was observed. Surgery was started only after the abdominal suspension and cardiotocography monitor had been adjusted and stable maternal hemodynamics and fetal heart rate had been confirmed.

Posterior lumbar discectomy was then performed. A 2.0 g herniated mass, including cartilaginous endplate fragments, was removed. The operative time was 51 minutes, the anesthesia time was two hours and 28 minutes, and the prone-position duration was one hour and 41 minutes. Fetal monitoring remained stable throughout the operation.

After emergence from anesthesia, the patient reported marked relief of leg pain. Bladder and bowel dysfunction improved gradually during the postoperative hospital stay. The visual analog scale score for leg pain improved from 10 preoperatively to 0 at one month after surgery. She was discharged home on postoperative day nine using a cane. Mild perineal sensory disturbance persisted. At 39 weeks of gestation, she delivered a healthy boy by cesarean section. At one year after surgery, she had no recurrence of pain and had normal urinary and bowel function, although mild perineal hypoesthesia remained. Early postoperative MRI was not performed because of marked clinical improvement. MRI at one year after surgery confirmed disappearance of the herniated mass.

## Discussion

This case shows that posterior lumbar discectomy in the prone position can be performed during late pregnancy when abdominal compression is avoided, maternal hemodynamics are maintained, and fetal monitoring is continued. The important point of this case is not the discectomy itself, but the practical method used to secure a safe prone position in a third-trimester pregnant patient.

LDH during pregnancy is usually managed conservatively. However, when bladder and bowel dysfunction or progressive neurological deficits occur, surgical indications are essentially the same as in non-pregnant patients. In the present case, bladder and bowel dysfunction developed during hospitalization, and urgent decompression was required. Delaying surgery because of pregnancy could have increased the risk of persistent neurological deficit.

Selection of the operative position is one of the main concerns in pregnant patients. Prone positioning is relatively easy in early pregnancy, but it becomes increasingly difficult as gestation progresses. In the third trimester, the lateral decubitus position is often preferred to reduce the risk of inferior vena cava compression [7]. Previous reports have included only a limited number of third-trimester patients who underwent lumbar disc herniation surgery in the prone position before delivery, and some late-pregnancy cases were treated in the prone position only after cesarean delivery [8]. In our review, descriptions of abdominal decompression and fetal monitoring during prone-position surgery before delivery were limited (Table 1).

**Table 1:** A review of reported cases of surgical intervention for lumbar disc herniation during pregnancy PP, prone position; LLP, left lateral position; RLP, right lateral position; N/A, unknown position; L, lumbar; S, sacral; PHL, partial hemilaminectomy.

Author	Age (years)	Gestational age (weeks)	Surgical position	Level	Operation method
Garmel et al. [8]	34	9	N/A	L5-S1	Discectomy
Han et al. [9]	32	11	PP	L4-L5	PHL, Discectomy
Curtin et al. [10]	37	11	N/A	L4-L5	Discectomy
Han et al. [9]	30	15	RLP	L5-S1	PHL, Discectomy
Brown et al. [11]	32	16	PP	L5-S1	Laminectomy and Discectomy
Abou-Shameh et al. [12]	34	19	PP	L4-L5	Discectomy
LaBan et al. [13]	36	20	N/A	L5-S1	Laminectomy and Discectomy
Brown et al. [11]	41	20	PP	L5-S1	Laminectomy and Discectomy
Brown et al. [11]	31	20	PP	S1	Laminectomy and Discectomy
Iyilikci et al. [5]	31	20	LLP	L5-S1	Discectomy
Garmel et al. [8]	29	24	N/A	L5-S1	Discectomy
Hakan et al. [14]	34	25	PP	L5-S2	Discectomy
Han et al. [9]	34	26	RLP	L4-L5	PHL, Discectomy
Garmel et al. [8]	28	30	N/A	L5-S1	Discectomy
Han et al. [9]	33	30	LLP	L4-L5	PHL, Discectomy
Kim et al. [15]	30	30	PP	L4-L5	Endoscopic discectomy
Han et al. [9]	30	32	LLP	L4-L5	PHL, Discectomy
Fahy et al. [16]	32	32	PP	L4-L5	Discectomy
This case	42	32	PP	L4-L5	Discectomy
Kathirgamanathan et al. [17]	34	33	LLP	L4-L5	Discectomy
Fahy et al. [16]	31	33	PP	L4-L5	Discectomy
Vougioukas et al. [6]	30	35	LLP	L4-L5	PHL, Discectomy

The lateral position has advantages in pregnant patients, especially for avoiding direct abdominal pressure. Endoscopic surgery can also be performed in the lateral position and may reduce soft-tissue damage and blood loss [15]. However, endoscopic discectomy requires specific equipment and experience and may not be suitable for every emergency case [18]. In contrast, posterior discectomy in the prone position is a standard procedure familiar to most spine surgeons. If a safe prone position can be obtained, it may allow rapid and reliable decompression.

Several approaches have been used to reduce aortocaval compression during surgery in late pregnancy, including the left lateral decubitus position, lateral uterine displacement, and prone positioning with the abdomen free. In our case, a four-point support table was used to support the chest and pelvis and to avoid direct abdominal pressure. However, the enlarged abdomen had no support in this setting. We therefore used elastic bandages and stockinettes to suspend the abdomen in a hammock-like manner. This reduced downward displacement of the gravid uterus while maintaining a small free space under the abdomen. It also allowed attachment of the CTG monitor. One practical point was that the stockinettes required firm fixation because complete muscle relaxation under general anesthesia caused more abdominal descent than expected. This detail may be important when reproducing this positioning method.

Anesthetic management was also essential. Maternal hypotension can reduce uteroplacental perfusion because uteroplacental circulation has limited autoregulation [19]. In this case, blood pressure decreased after prone positioning, probably because of the combined effects of general anesthesia and the prone position [20]. A continuous norepinephrine infusion was initiated and titrated to restore blood pressure to the target range. The four-point support operating table allowed continuous cardiotocographic monitoring from induction onward, providing real-time data for vasopressor titration during the hypotensive episode and for ongoing fetal assessment. In this case, continuous CTG monitoring allowed surgery to proceed despite the inability to perform a pre-induction simulation.

The feasibility of prone-position surgery in late pregnancy cannot be determined by gestational age alone. It should be assessed according to maternal body habitus, fetal status, urgency of neurological symptoms, and the institutional ability to perform emergency cesarean delivery if needed. In the present case, the patient was at 32 weeks of gestation, and emergency cesarean delivery was available as a backup option.

This case has limitations. It is a single case, and the safety of this method cannot be generalized to all pregnant patients. Maternal body habitus, gestational age, fetal status, urgency of neurological symptoms, and the experience of the surgical and anesthesia teams must be considered. This approach should be considered only in carefully selected patients after multidisciplinary discussion and in institutions where emergency obstetric intervention is available.

## Conclusions

Prone-position lumbar surgery during late pregnancy requires attention to positioning before anesthesia is induced. The abdomen should be kept free from compression, and fetal monitoring should be continued when feasible. Maternal blood pressure should be checked closely after making the patient prone. A plan for emergency delivery should be shared before surgery. The choice of position should be made by the surgical, anesthesia, and obstetric teams. This case describes one such practical approach for a similar emergency.
